# Ultrasound findings in Kaposi sarcoma patients: overlapping sonographic features with disseminated tuberculosis

**DOI:** 10.1186/s13089-023-00323-8

**Published:** 2023-06-01

**Authors:** Michaela Aurelia Maria Huson, Tapiwa Kumwenda, Joe Gumulira, Ethel Rambiki, Claudia Wallrauch, Tom Heller

**Affiliations:** 1 Department of Internal Medicine, Radboud University Medical Center, Nijmegen, The Netherlands; 2 Lighthouse Clinic, Kamuzu Central Hospital, Area 33, Mzimba Street, 106, Lilongwe, Malawi; 3 International Training and Education Center for Health, University of Washington, Seattle, WA USA

**Keywords:** Ultrasound, Kaposi sarcoma, HIV, FASH

## Abstract

**Background:**

Focused Assessment with Sonography for HIV-associated TB (FASH) is a diagnostic tool for extra-pulmonary tuberculosis (TB) in symptomatic patients with advanced HIV. As Kaposi’s sarcoma (KS) is also prevalent in this patient population, changes due to KS may mimic TB findings and clinical interpretation of target FASH findings can be challenging. We aimed to describe sonographic findings in patients with KS.

**Methods:**

We performed a prospective observational study at Lighthouse clinic at Kamuzu Central Hospital, Lilongwe, Malawi, in consecutive patients with newly diagnosed KS, without known diagnosis of TB, referred for paclitaxel treatment. All patients underwent FASH and abdominal ultrasound to assess for effusions and changes in liver and spleen, as well as systematic sonographic assessment for lymphadenopathy.

**Results:**

We included 30 patients. We found inguinal lymph nodes using ultrasound in 20 patients; in 3 (10%) additionally abdominal lymph nodes were found. Pathological effusions were seen in eight patients (27%): pericardial effusion in one (3%), pleural effusion in six (20%) and ascites in four (13%) patients. We found focal spleen lesions in three (10%) patients. Most of these lesions were echogenic, but in one patient, we saw hypoechoic lesions with an echogenic center. In three (10%) patients an unusual “sponge-like pattern” of the splenic vasculature was found. Six (20%) patients had echogenic focal lesions in the liver resembling hemangiomas, individual lesions showing a hypoechoic center. In two patients echogenic portal fields were seen.

**Conclusions:**

The majority of patients with newly diagnosed KS demonstrate sonographic features of disease, predominantly lymphadenopathy. Effusions were observed in a significant minority, as well as focal lesions in liver or spleen, which commonly resemble hemangiomas, but hypoechoic lesions were also observed and can easily be mistaken for extra-pulmonary TB. A 'sponge-like pattern' of the spleen should not be confused with micro-abscesses. In conclusion, this case series illustrates the diverse nature of ultrasound features in patients with KS, which can be difficult to distinguish from other opportunistic diseases, including TB.

**Supplementary Information:**

The online version contains supplementary material available at 10.1186/s13089-023-00323-8.

## Background

Ultrasound is a versatile diagnostic tool, which has been classified by the World Health Organization as an essential diagnostic test [1]. In settings endemic for HIV and tuberculosis (TB), the Focused Assessment with Sonography for HIV/TB (FASH) has been developed to assess patients for signs of extra-pulmonary TB [2]. The protocol includes an assessment of pericardial and pleural effusions, ascites, abdominal lymphadenopathy and splenic micro abscesses. It is widely implemented in Sub-Saharan Africa, due to its high clinical relevance, availability at the bedside and ease to learn for non-expert sonographers [3, 4]. FASH is aimed at patients with HIV who present with symptoms, such as cough, weight loss, fever and night sweats, which are all consistent with extra-pulmonary tuberculosis. However, other HIV-related pathologies can cause similar symptoms, including disseminated Kaposi sarcoma (KS), as well as a wide variety of other opportunistic diseases. Ultrasound findings may overlap between extra-pulmonary TB and KS and differentiating between the different conditions can be challenging in clinical practice.

Few reports exist on ultrasound findings in KS patients, especially from African settings, were most patients are seen. HIV-associated KS usually presents on the skin, the lining of the mouth, viscera (e.g., lungs and gastrointestinal tract), and lymph nodes. A recent review on imaging findings in KS reported masses, nodules, thickening of the bronchovascular tree, ground glass opacities and pleural effusions in intra-thoracic KS [5]. Abdominal KS can present with hepato- or splenomegaly, and nodules in the liver and more rarely, in the spleen, which appear hyperechoic on ultrasound [5]. Small periportal nodules causing increased periportal echogenicity have also been described [6]. Gastrointestinal KS may result in wall thickening, ascites and lymphadenopathy. Genitourinary KS may sometimes cause outflow obstruction and urinary retention [5]. Ultrasound findings in KS thus include effusions (pericardial, pleural, ascites), lymphadenopathy, and hyperechoic lesions in liver or spleen.

We here aimed to describe ultrasound findings in African patients with proven KS. Our findings will help to determine signs that may overlap between patients with extra-pulmonary TB and signs are that more specific to TB. These findings can aid the clinician performing a FASH examination in interpreting target FASH findings and differentiating between KS and extra-pulmonary TB in a HIV/TB endemic setting, but may also be of broader use in the assessment and follow-up of patients with (suspected) KS.

## Methods

A prospective observational study was performed at Lighthouse clinic at Kamuzu Central Hospital, Lilongwe, Malawi. Consecutive patients with newly diagnosed KS, confirmed by histology of skin biopsies who were referred for paclitaxel treatment, were included. None of the patients had a known diagnosis of TB or suggestive clinical, urine-LAM, or chest X-ray findings. Demographic and clinical data were extracted using a standard case report form and saved in an anonymized database. As part of their routine pre-treatment care, patients were examined and staged according to tumor extension (T0 vs. T1) [7] and underwent laboratory investigations (CD4 count, full blood count, liver function tests and creatinine) as well as a CXR according to local protocols. All patients underwent systematic sonographic assessment using a b/w-ultrasound scanner (DP-30, Mindray, China) with convex (35C50EA) and high-frequency linear (75L38EA) probe assessing for lymphadenopathy (axillary, nuchal, abdominal and inguinal), effusions (pleural, pericardial, ascites), and changes in liver and spleen. Spleen and superficial areas of the liver were routinely scanned with the linear probe. Data were analyzed using descriptive statistics. All data were collected as part of routine clinical care; approval was granted by the Malawi National Health Science Research Committee for the collection and use of clinical and programmatic data (NHSRC Protocol #2812) used in this report.

## Results

Baseline characteristics and ultrasound features are summarized in Table 1. From July 2021 to July 2022, we included 30 patients, 22 male and 8 female, with a median age of 39 [interquartile range (IQR) 34–45] years. All except one were HIV positive; 12 had not yet started ART, 10 had started recently (< 6 months) and 7 were taking ART for longer than 6 months. CD4 T-cell count results were available for 27; the median was 192 cells/mm^3^ (IQR 109–300). Median Body Mass Index was 21.4 kg/m^2^ (IQR 20.4–23.5); median hemoglobin 10.2 g/dL. 26% had severe anemia below 9 g/dl, all of which were normocytic. Other laboratory investigations (WBC, platelets, ALT, total bilirubin and creatinine) were normal in all cases.

**Table 1:** Baseline characteristics and ultrasound features of 30 patients with Kaposi's sarcoma treated al Lighthouse clinic

Baseline characteristics	
Sex, male/female, n (%)	22/8 (73/27)
Median age (IQR)	39 (34–45)
Median body mass index (kg/m^2^) (IQR)	21.4 (20.4–23.5)
HIV positive, n (%)	29 (97)
CD4 T-cell count (cells/mm^3^)^a^	192 (109–300)
ART status	
ART not yet started, number (%)	12 (40)
ART < 6 months, n (%)	10 (33)
ART > 6 months, n (%)	7 (23)
KS staging	
Skin lesions T0, n (%)	10 (33)
Skin lesions T1, n (%)	20 (67)
Oral lesions, n (%)	17 (60)
Leg edema, n (%)^b^	20 (67)
CXR suggestive for KS lung involvement, n (%)^c^	10 (40)

Ultrasound features (n, %)^d^	
Lymphadenopathy	20 (67)
Inguinal lymphadenopathy	*20 (67)*
Abdominal lymphadenopathy	*3 (10)*
Axillary lymphadenopathy	*1 (3)*
Effusions	8 (27)
Pleural effusion	*6 (20)*
Ascites	*4 (13)*
Pericardial effusion	*1 (3)*
Echogenic focal liver lesions	6 (20)
Splenomegaly^e^	3 (10)
Focal spleen lesions	3 (10)
Sponge pattern of splenic vasculature	3 (10)
Echogenic portal fields	2 (7)
Hepatomegaly	1 (3)

*n* number, *IQR* interquartile range, *ART* antiretroviral therapy

^a^Data available for 27 patients

^b^Including one patient with concomitant periorbital edema

^c^Data available for 25 patients

^d^All percentages are presented as the percentage of the total number of patients included

^e^All were mild cases of splenomegaly (13–14 cm)

All patients showed typical KS skin changes; the extent of the KS skin involvement was graded “limited” (T0) in 10 (33%) and “extensive” (T1) in 20 (67%); 20 (67%) patients had significant leg edema; oral involvement was seen in 17 (57%) patients. A chest X-ray was available for 25 patients; it was suggestive of KS lung involvement (effusions or streaky infiltrates in lower lung fields) in 10 (40%).

**Fig. 1:**
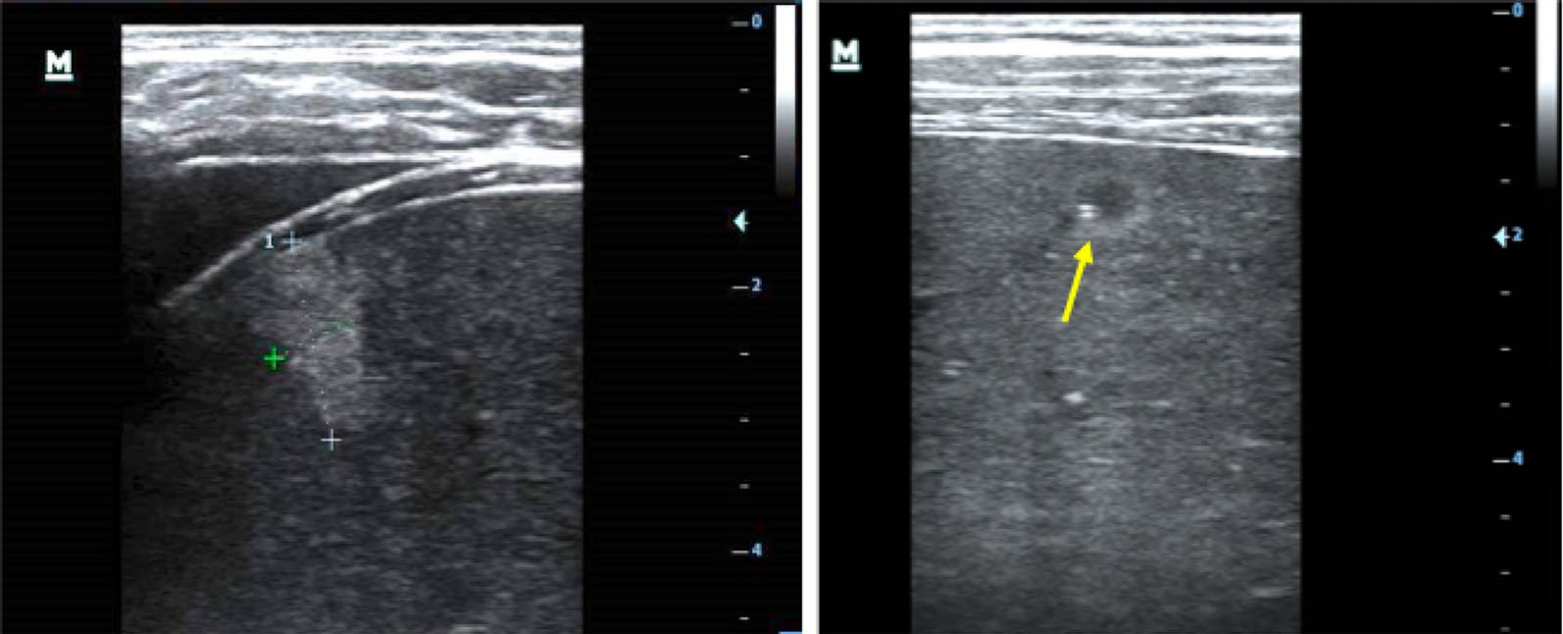
Focal liver lesions resembling **a** hemangioma (39-year-old male, CD4 T-cell count 192 cells/mm^3^, on ART < 6 months) and **b** individual lesions with bulls’ eye appearance (arrow; 46-year-old male, CD4 T-cell count 103 cells/mm^3^ l, on ART < 6 months)

**Fig. 2:**
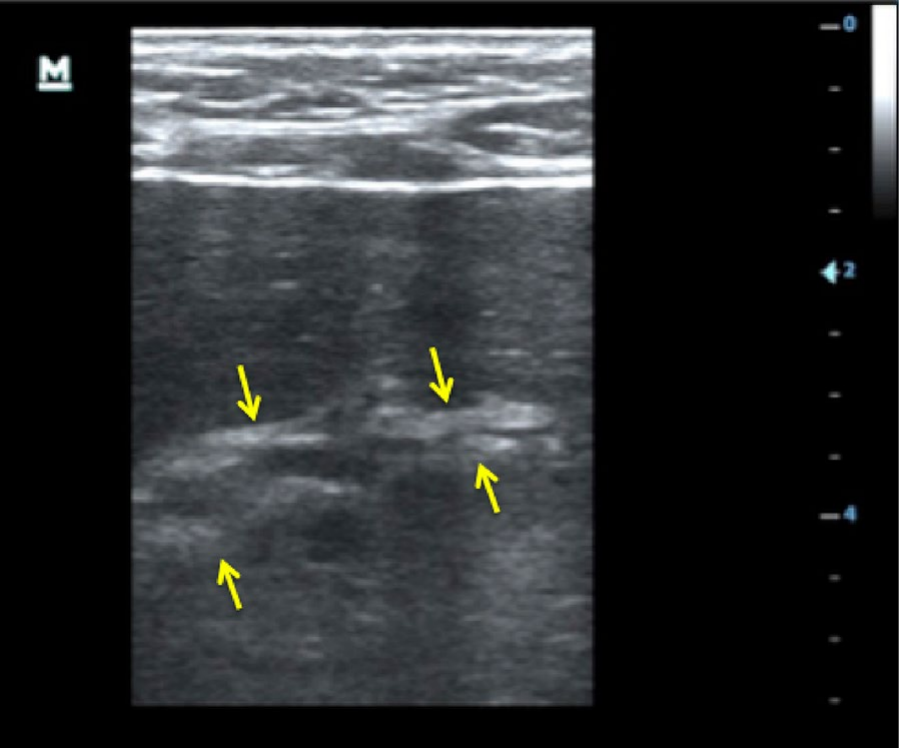
Echogenic portal fields (same patient as Fig. 1b)

**Fig. 3:**
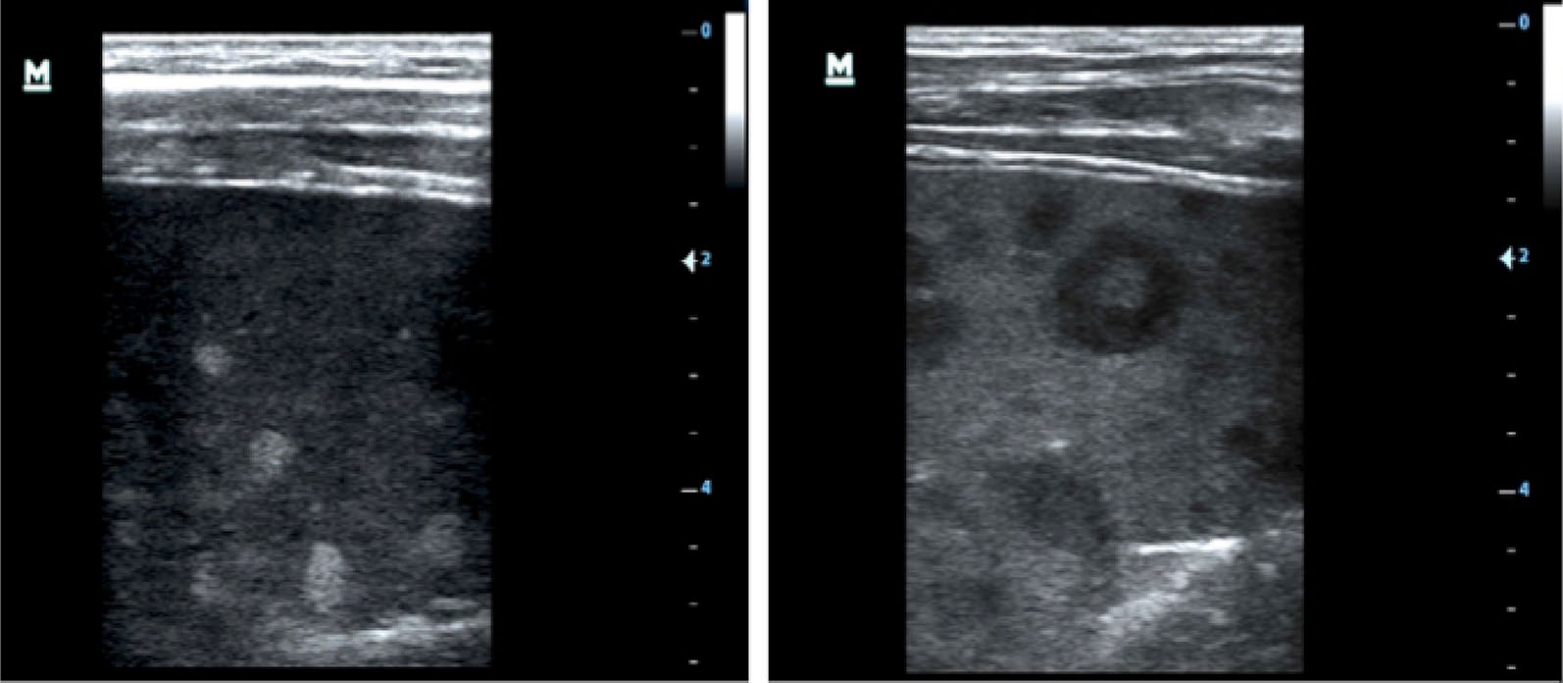
**a** Hyperechoic splenic lesions (46-year-old male, CD4 T-cell count 103 cells/mm^3^, started ART less than 6 months) and **b** hypoechoic splenic lesions with a hyperechoic center (39-year-old male, CD4 T-cell count 93 cells/mm^3^, not on ART)

**Fig. 4:**
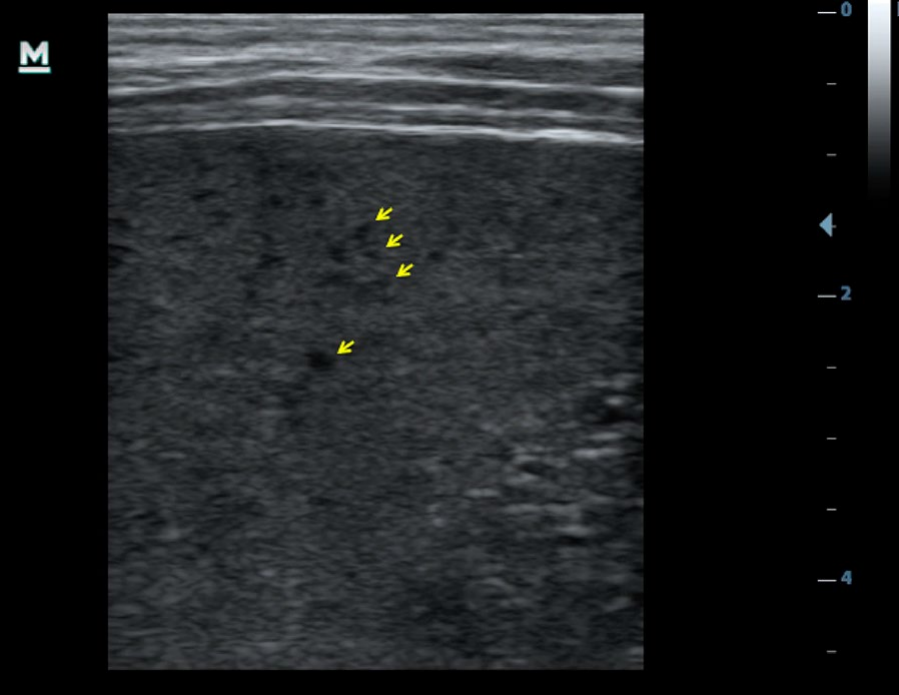
Tiny hypoechoic lesions: Sponge-like pattern of the spleen (better visible in the supp. video; 34-year-old female, CD4 T-cell count 124 cells/mm^3^, not on ART)

Using ultrasound, lymphadenopathy was detected in 20 patients (67%), all of whom had inguinal lymphadenopathy. Three patients (10%) additionally had abdominal lymph nodes, and one patient additionally had axillary lymph nodes. Any pathological effusion was seen in eight patients (27%): pleural effusion in six (20%), ascites in four (13%) and pericardial effusion in one (3%). Six patients (20%) had echogenic focal lesions in the liver, mostly resembling hemangiomas (Fig. 1). In one patient some lesions had central hypoechoic areas (bulls’ eye appearance, Fig. 1); in two patients echogenic portal fields were seen (Fig. 2). Three patients had mild splenomegaly (length 13–14 cm). Focal spleen lesions were detected in 3 (10%) patients; these were mainly echogenic lesions and, in one patient, hypoechoic lesions with a hyperechoic center (Fig. 3) were seen. In three patients (10%) an unusual “sponge-like pattern” of the splenic vasculature was found, showing very small (1.5–2 mm) hypoechoic lesions with a reticulo-nodular distribution (Fig. 4, Additional file 1: Video clip).

## Conclusions and discussion

In this case series, we systematically describe ultrasound features prevalent in African patients with KS. The most common findings were enlarged lymph nodes and effusions. In liver and spleen, focal lesions were observed; these were mostly hyperechoic, resembling hemangiomas. Mixed hyper-hypoechoic lesions (target- and bull’s eye lesions) were also seen. In three patients, we documented a “sponge-like pattern” of spleen tissue. Findings in spleen and liver were often faint and better visible using the linear probe; the use of the high-frequency probe likely added to the relatively high proportion of solid organ involvement documented in our series. The sonographic findings identified resemble those previously described in pictorial reviews [5, 6]; to our knowledge, the frequency of the various findings has not been previously described.

Ultrasound findings of the FASH scan—effusions, abdominal lymphadenopathy and focal spleen lesions—are frequently used to aid the diagnosis of disseminated TB in resource-limited high-prevalence settings. It is, therefore, important to discuss the overlap with findings in KS patients to prevent misdiagnosis.

The enlarged lymph nodes resemble those of disseminated TB, although the inguinal localization is more suggestive of KS. Nevertheless, abdominal lymphadenopathy due to KS can also be found and can be mistaken for a sign of disseminated TB. The KS-associated effusions in our patients showed no differentiating patterns and could also be mistaken for a sign of disseminated TB. In the interpretation of the findings, it should be noted that KS-associated effusions and intra-abdominal lymphadenopathy, have a broad differential diagnosis, including extra-pulmonary TB, disseminated *Mycobacterium avium* complex (MAC) infection, HIV lymphadenopathy, Hodgkin- or non-Hodgkin lymphoma, and other human herpes virus 8 (HHV-8)-associated illnesses, such as multicentric Castleman disease (MCD) and primary effusion lymphoma [8, 9].

The sonographic lesions of KS in spleen and liver are more pathognomonic. Hyperechoic lesions in a patient with HIV, a low CD4 T-cell count and systemic symptoms, are highly suggestive of KS and are unlikely to be mistaken. They should prompt an immediate (re-)evaluation of the entire skin, especially of legs and groins, and oral mucosa to search for KS skin manifestations. We also identified hypoechoic splenic lesions with a hyperechoic center (target lesions), and these can be difficult to distinguish form micro-abscesses when they are small and the examiners less experienced.

Finally, we found a sponge-like pattern of the spleen, which has only recently been described in adult HIV positive patients [10]. This sponge pattern can be mistaken for spleen micro-abscesses, although the hypoechoic structures are generally smaller [1–2 mm] then lesions caused by TB (> 5 mm). Our data presented here suggest that the sponge pattern may be seen in about 10% of KS patients, although our absolute patient number was small (95% confidence interval: 0–21%). The pattern was originally described in association with a variety of opportunistic illnesses, including KS, disseminated MAC infection, pneumococcal infection, Hodgkin lymphoma and MCD as well as with HIV itself—still almost half of the reported cases were associated with HHV-8 associated diseases, such as KS or MCD [10]. A recent radiological review states that KS tends to “surround the arteries of the Malpighian corpuscles and has a stringy appearance”[4]—which could explain the sponge pattern in ultrasound—but actual histo-pathological evidence for this in newer literature is scarce. Still, an autopsy series from the early 1980s before HIV testing became available found KS involvement in 32 of 44 spleens of patients who died with clinical AIDS and reported that “in the spleen the perivascular white pulp is most commonly and extensively involved” [11].

In summary, our results highlight the importance to place ultrasound findings in clinical context and perform a detailed history (e.g., detailed symptoms, exposure to TB) and physical examination to assess for KS skin lesions. Only 1–2% of KS patients present with enlarged LN without skin lesions [12], thus in the vast majority of cases skin changes will be found if thoroughly looked for. All patients in our cohort showed typical skin changes. Our findings also underline that even in resource-limited settings a pathological confirmation is desirable for many diseases due to overlapping findings of various differentials; nevertheless this is rarely achievable and clinical observation of treatment response remains a mainstay of making diagnoses in our setting.

Our study was limited by the fact that it was performed in a single center with a relatively small sample size. In addition, while the diagnosis of KS was made clinically and supported histologically, we were not able to entirely rule-out diagnosis of other opportunistic infections or of concomitant TB (beyond clinical assessment, chest X-ray and urine LAM tests). Our clinic setting was also not conducive to sonographic follow-up of patients; therefore, we cannot report on the changes of the findings under treatment. However, to the best of our knowledge, this is the first systematic African case series providing estimates of frequency of sonographgic findings prevalent in Kaposi sarcoma. Overall ultrasound findings in KS seem to be more frequent than previously thought, especially if linear transducers are used to evaluate solid organs.

## Conclusion

This case series demonstrates a diverse range of ultrasound features in patients with KS. Ultrasound can be a valuable tool in the diagnostic work-up of patients with (suspected) TB but also with KS. However, it can be difficult to differentiate between opportunistic diseases based on ultrasound features and careful clinical assessments, especially of skin on the legs, groins and of the palate, combined with pathology investigations if available, are required to place the ultrasound findings in the clinical context.

## Supplementary Information

**Additional file 1: Video clip**: Sponge-like pattern of the spleen (34-year old female, CD4 T-cell count 124 cells/mm^3^, not on ART).

## Data Availability

Not applicable.

## Abbreviations

HIV: Human immunodeficiency virus
FASH: Focused assessment with sonography for HIV-associated TB
IQR: Interquartile range
KS: Kaposi’s sarcoma
MAC: *Mycobacterium avium* complex
TB: Tuberculosis
